# Synthesis and structure of 2-oxo-2*H*-chromen-4-yl 4-bromo­benzoate: work carried out as part of the CNRS AFRAMED project

**DOI:** 10.1107/S2056989025000246

**Published:** 2025-02-07

**Authors:** Valentin Bationo, Konan René Kambo, Brahima Sorgho, Charles Bavouma Sombié, El Walda Thiam, Rasmané Semdé, Abdoulaye Djandé, Claude Lecomte, Emmanuel Wenger

**Affiliations:** a Laboratory of Molecular Chemistry and Materials (LC2M), University Joseph KI-ZERBO, 03 BP 7021 Ouagadougou 03, Burkina Faso; b Laboratory of Environmental Science and Technology University Jean Lorougnon GUEDE of Daloa BP 150 Daloa Côte d’Ivoire; c Laboratory of Drug Development, Center of Training, Research and Expertise in Pharmaceutical Sciences (CFOREM), University Joseph KI-ZERBO, 03 BP 7021, Ouagadougou 03, Burkina Faso; d Laboratory of Environment, Health and Society, University of Nouakchott, BP 880-Nouakchott, Mauritania; e CRM2, CNRS-Université de Lorraine, Vandoeuvre-lès-Nancy CEDEX BP 70239, France; University of Aberdeen, United Kingdom

**Keywords:** crystal structure, hydrogen bonds, Hirshfeld surface analysis, coumarin

## Abstract

In the title compound, C_16_H_9_BrO_4_, the dihedral angle between the chromen-2-one ring system (r.m.s. deviation = 0.006 Å) and the bromo­benzene ring is 10.29 (6)°. In the crystal, the mol­ecules are connected through C—H⋯O hydrogen bonds and π–π stacking inter­actions. According to a Hirshfeld surface analysis, H⋯H (22.4%), O⋯H/H⋯O (23.6%) and C⋯H/H⋯C (21%) inter­actions are the most significant contributors to the crystal packing.

## Abstract 2

In the title compound, the dihedral angle between the chromen-2-one ring system and the bromo­benzene ring is 10.29 (6)°. In the crystal, the mol­ecules are connected through C—H⋯O hydrogen bonds and π–π stacking inter­actions.

## 1. AFRAMED and chemical context

This work was carried out as part of the CNRS AFRAMED project, which aims to train African Partners (young lecturers with permanent positions) in X-ray diffraction and provide regional laboratories, which serve as focal points to assist their colleagues for remote measurements (Abdel-Aal *et al.*, 2023 ▸). Coumarin derivatives remain one of our research priorities due to their versatile range of activities, such as anti­coagulant, anti-inflammatory, anti­viral, anti­microbial, anti­cancer, anti­oxidant (Todorov *et al.*, 2023 ▸), anti-glaucoma (Ziki *et al.*, 2023 ▸) and anti-Parkinsonian effects (Kambo *et al.*, 2024 ▸). Here we report one result of this training: the synthesis, crystal structure and Hirshfeld surface analysis of the title coumarin derivative, C_16_H_9_BrO_4_ (**I**).
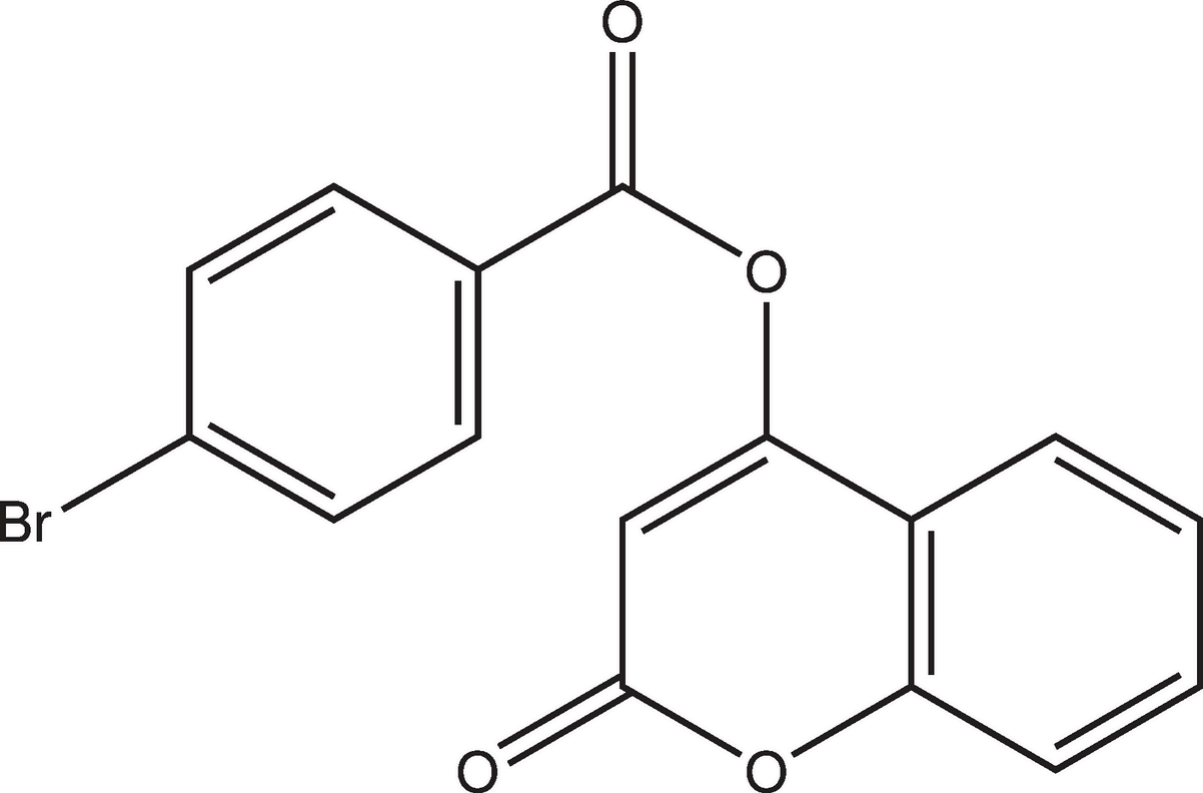


## 2. Structural commentary

**Figure 1:**
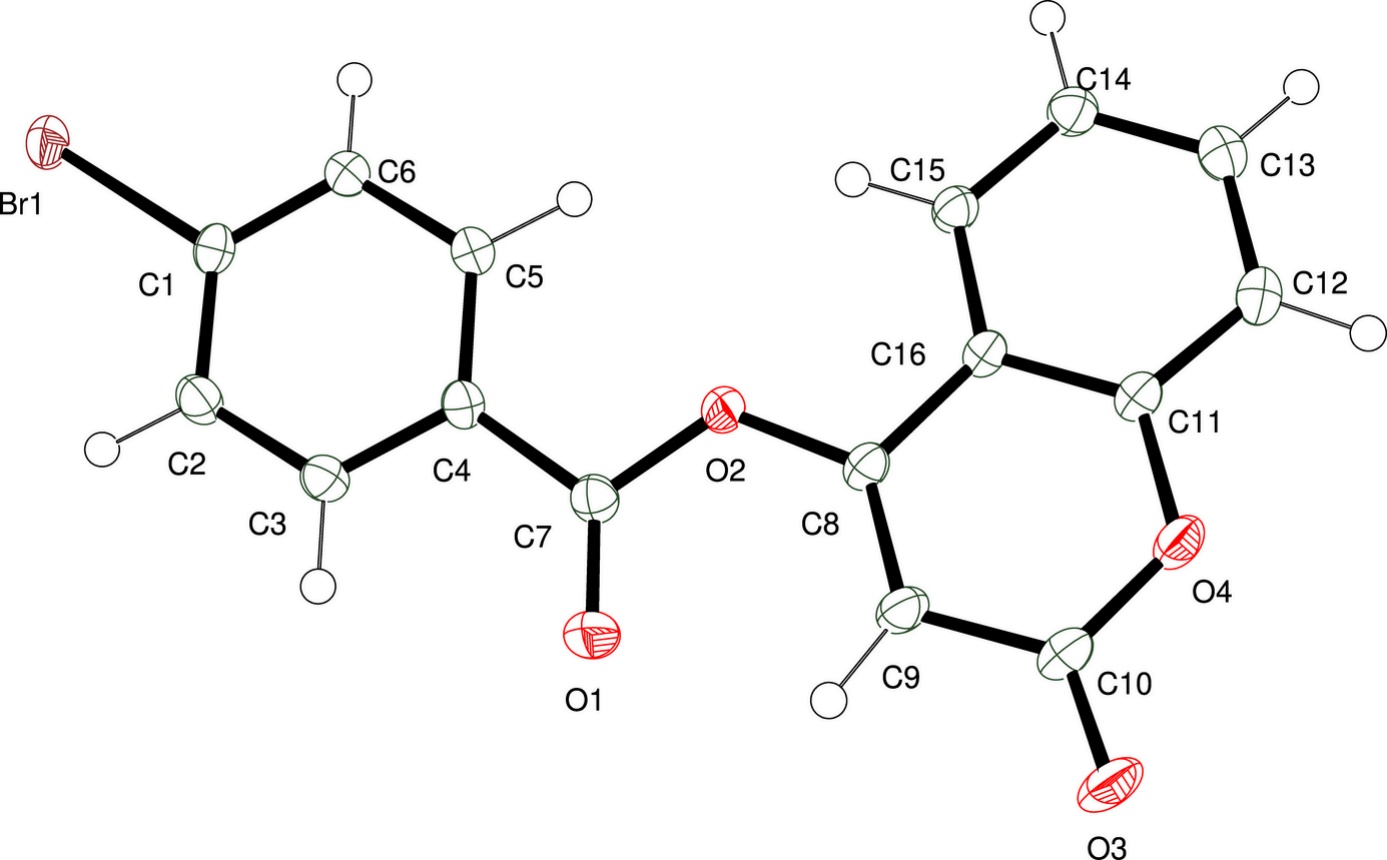
The mol­ecular structure of (**I**) with displacement ellipsoids drawn at the 50% probability level.

Compound (**I**) crystallizes in the ortho­rhom­bic space group *P*2_1_2_1_2_1_ with one mol­ecule in the asymmetric unit (Fig. 1 ▸). The side chain is titled with respect to the chromen-2-one ring system with torsion angles C9—C8—O2—C7 = −12.3 (4)° and C16—C8—O2—C7 = 169.9 (2)°. As expected, the chromen-2-one ring (C8—H16) system is almost planar, with a maximum deviation from the mean plane of 0.030 (2) Å for atom O3. The dihedral angle between this coumarin ring and the C1–C6 phenyl group in the 4-bromo­benzoate moiety is 10.29 (6)°. An inspection of the bond lengths shows that there is a slight asymmetry of the electronic distribution around the pyrone ring, as shown by the differences between C8—C9 [1.350 (3) Å] and C9—C10 [1.452 (4) Å]. This suggests that the electron density is preferentially located in the formal C8=C9 double bond of the pyrone ring, as seen in other coumarin derivatives (Gomes *et al.*, 2016 ▸; Ouédraogo *et al.*, 2018 ▸). One intra­molecular short contact exists between the C9—H9 methine group and atom O1 (Table 1 ▸).

## 3. Supra­molecular features and Hirshfeld surface analysis

**Figure 2:**
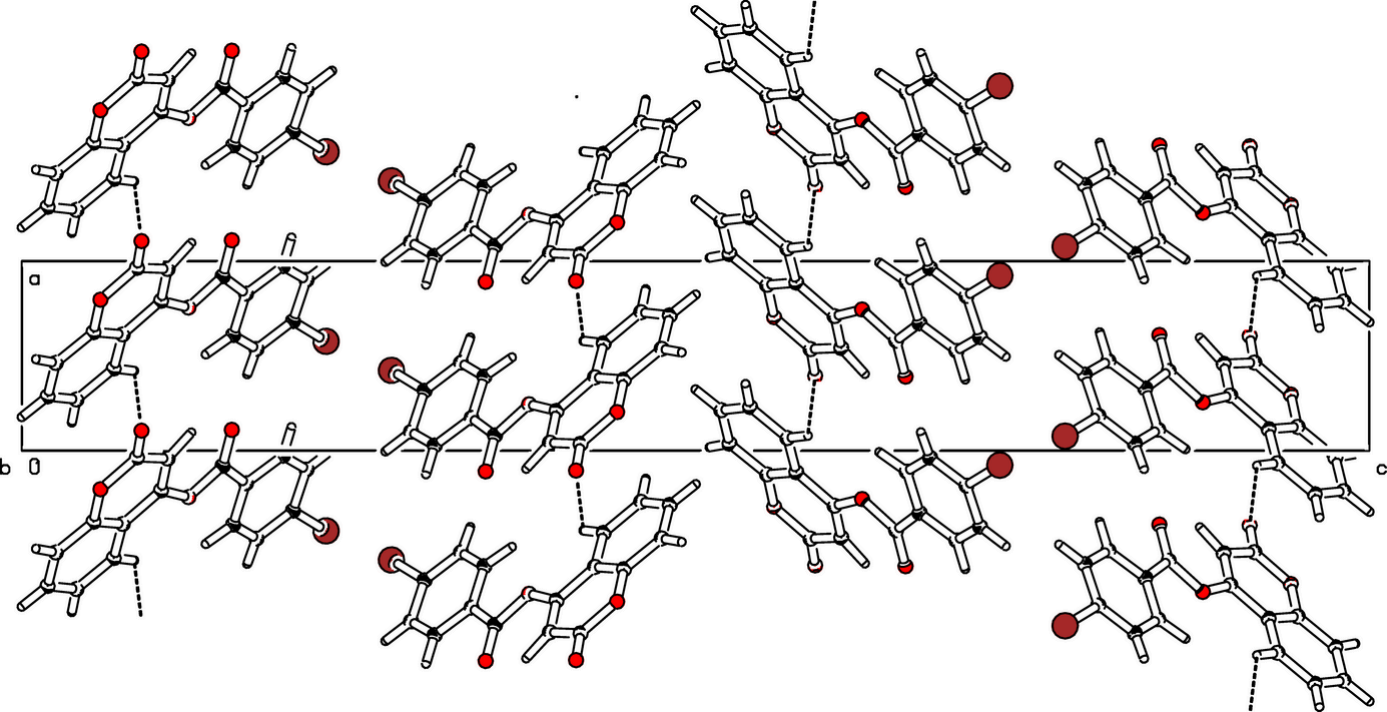
The unit-cell packing of (**I**) showing a hydrogen-bonded [1
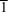
0] chain: dashed lines indicate hydrogen bonds. H atoms not involved in hydrogen-bonding inter­actions have been omitted for clarity.

In the crystal of (**I**), a weak C15—H15⋯O3 hydrogen bond links the mol­ecules into [1
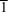
0] chains (Table 1 ▸, Fig. 2 ▸). The three-dimensional architecture is further consolidated by aromatic π-stacking inter­actions: the *Cg*1⋯*Cg*3(1 + *x*, *y*, *z*) separation is 3.8770 (19) Å, where *Cg*1 and *Cg*3 are the centroids of the C8–C10/O4/C11/C16 and C11–C16 rings, respectively.

**Figure 3:**
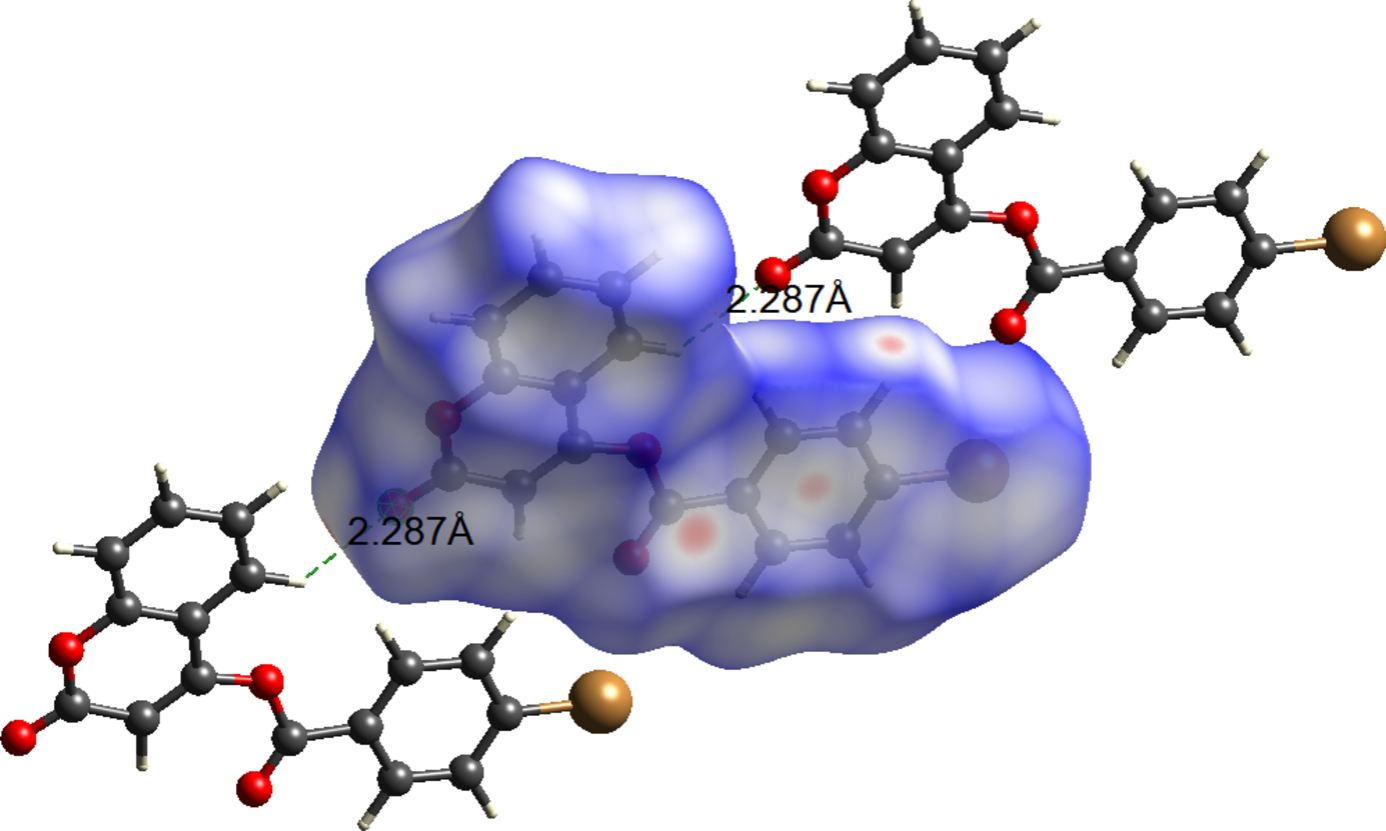
The Hirshfeld surface of (**I**) mapped over *d*_norm_. Dotted lines (magenta) represent hydrogen bonds.

**Figure 4:**
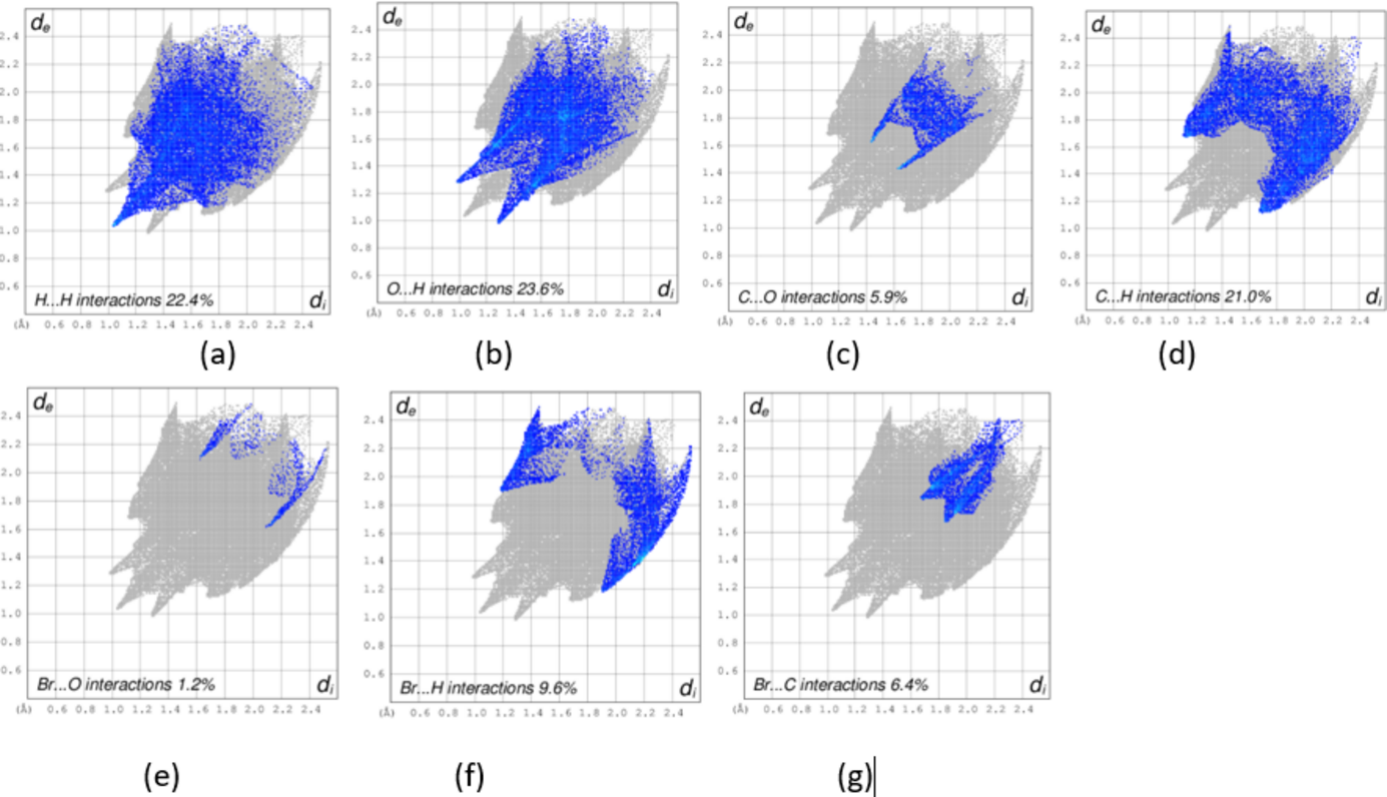
Fingerprint plots of (**I**): (*a*) H⋯H, (*b*) O⋯H, (*c*) C⋯O, (*d*) C⋯H, (*e*) Br⋯O, (*f*) Br⋯H and (*g*) Br⋯C: *d*_i_ is the closest inter­nal distance from a given point on the Hirshfeld surface and *d*_e_ is the closest external contact.

The inter­molecular inter­actions in (**I**) were further qu­anti­fied by Hirshfeld surface analysis (Fig. 3 ▸) using *CrystalExplorer* (Spackman *et al.*, 2023 ▸). The inter­actions mentioned above are confirmed by the two-dimensional fingerprint plots for (**I**) (Fig. 4 ▸). The greatest contributions are from O⋯H/H⋯O (23.6%), H⋯H (22.4%) and C⋯H/H⋯C (21%) while the Br⋯H/H⋯Br and Br⋯C/C⋯Br contacts contribute 9.6 and 6.4%, respectively.

## 4. Database survey

A search of the Cambridge Structural Database (CSD, version 5.43; update 3, September 2022; Groom *et al.*, 2016 ▸) for structures having a coumarin motif similar to (**I**) returned thirteen hits, including (7-chloro-2-oxo-2*H*-chromen-4-yl)methyl­dimethyl­carbamodi­thio­ate (CSD refcode XUFGOW; Kavitha *et al.*, 2015 ▸); (6-bromo-2-oxo-2*H*-chromen-4-yl)meth­yl di­ethyl­carbamodi­thio­ate (NUZJOJ; Vinduvahini *et al.*, 2016 ▸); (5,7-dimethyl-2-oxo-2*H*-chromen-4-yl)methyl morpholine-4-carbodi­thio­ate (UDOGIF01; Anitha *et al.*, 2016 ▸); (7-fluoro-2-oxo-2*H*-chromen-4-yl)methyl morpholine-4-carbodi­thio­ate (UYVEE; Anitha *et al.*, 2015 ▸); (7,8-dimethyl-2-oxo-2*H*-chromen-4-yl)methyl piperidine-1-carbodi­thio­ate (NAGWAW; Ravi *et al.*, 2016 ▸); methyl 2-[(2-oxo-2*H*-1-benzo­pyran-4-yl)amino]­benzoate (DIWPAE; Hollauer *et al.*, 2023 ▸). In 2-oxo-2*H*-chromen-4-yl 4-(dimetyl­amino)­benzoate (AYOXAO; Abou *et al.*, 2011 ▸), the benzoate ring is oriented at a dihedral angle of 43.43 (6)° with the chromene ring system while in 2-oxo-2*H*-chromen-4-yl 4-*tert*-butyl­benzoate (GARHAK; Abou et *et al.*, 2012*b* ▸), the benzene ring of the benzoate group is oriented at a dihedral angle of 60.70 (7)° with the chromene ring system. In 2-oxo-2*H*-chromen-4-yl 4-meth­oxy­benzoate (PECVUQ; Abou *et al.*, 2012*a* ▸), the chromen-2-one ring and the 4-meth­oxy­benzoate side chain are inclined to one another at a dihedral angle of 69.82 (9)° and in 2-oxo-2*H*-chromen-4-yl 4-methyl­benzoate (AFOQET; Abou *et al.*, 2013 ▸), the chromene-2-one ring and the 4-methyl­benzoate side chain are inclined to one another at a dihedral angle of 64.79 (10)° in one mol­ecule and 88.3 (1)° in the other. In 2-oxo-2*H*-chromen-4-yl propionate (AGAREH; Bibila Mayaya Bisseyou *et al.*, 2013 ▸), the 2-oxo-2*H*-chromene ring system and the non-H atom of the 4-substituent all lie on a crystallographic mirror plane. In 2-oxo-2*H*-1-benzo­pyran-4-yl 3,3-di­methyl­butano­ate (JOMHUS; Bationo *et al.*, 2024*a* ▸), the 2-oxo-2*H*-1-benzo­pyran-4-yl 3,3-di­methyl­butano­ate the coumarin ring system is oriented at a dihedral angle of 56.24 (18)° with the butano­ate moiety. In 2-oxo-2*H*-chromen-4-yl penta­noate (PONXUP; Bationo *et al.*, 2024*b* ▸), the dihedral angle between the coumarin ring system and the penta­noate is 36.26 (8)°.

## 5. Synthesis and crystallization

To a solution of 4-bromo­benzoyl chloride (6.2 mmol, 1.35 g) in dried tetra­hydro­furn (30 ml) were added dried tri­ethyl­amine (3 molar equivalents, 2.6 ml) and 4-hy­droxy­coumarin (6.17 mmol, 1.00 g) in small portions over 30 min. The mixture was then refluxed for 4 h and poured into 40 ml of chloro­form. The solution was acidified with dilute (5%) hydro­chloric acid until its discoloration was complete. The organic layers were extracted, concentrated under vacuum until a slight cloudiness was obtained and left in an ice bath. The resulting crude product was filtered off with suction, washed with petroleum ether and purified by recrystallization from a chloro­form–hexane solvent mixture: yield 71%.

Colorless crystals of (**I**) suitable for data collection were obtained by recrystallization from acetone solution. The melting point was measured in an open capillary with a Cole–Parmer STUART MP. 800D Series-Melting S apparatus and is thus uncorrected, m.p. 423–425 K.

## 6. Refinement

Crystal data, data collection and structure refinement details are summarized in Table 2 ▸. Hydrogen atoms were located in difference-Fourier maps and their positions and *U*_iso_ values were freely refined.

## Supplementary Material

Crystal structure: contains datablock(s) I, global. DOI: 10.1107/S2056989025000246/hb8116sup1.cif

Structure factors: contains datablock(s) I. DOI: 10.1107/S2056989025000246/hb8116Isup2.hkl

Supporting information file. DOI: 10.1107/S2056989025000246/hb8116Isup3.cml

CCDC reference: 2416578

Additional supporting information:  crystallographic information; 3D view; checkCIF report

## Figures and Tables

**Table 1:** Hydrogen-bond geometry (Å, °)

*D*—H⋯*A*	*D*—H	H⋯*A*	*D*⋯*A*	*D*—H⋯*A*
C9—H9⋯O1	1.00 (4)	2.26 (4)	2.788 (3)	111 (3)
C15—H15⋯O3^i^	0.97 (3)	2.36 (4)	3.079 (3)	131 (3)

Symmetry code: (i) 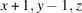.

**Table 2:** Experimental details

Crystal data	
Chemical formula	C_16_H_9_BrO_4_
*M*_r_	345.14
Crystal system, space group	Orthorhombic, *P*2_1_2_1_2_1_
Temperature (K)	100
*a*, *b*, *c* (Å)	5.4003 (11), 6.367 (2), 38.404 (8)
*V* (Å^3^)	1320.4 (6)
*Z*	4
Radiation type	Mo *K*α
μ (mm^−1^)	3.13
Crystal size (mm)	0.27 × 0.21 × 0.13

Data collection	
Diffractometer	Bruker D8 Venture
Absorption correction	Multi-scan (*SADABS*; Krause *et al.*, 2015 ▸)
*T*_min_, *T*_max_	0.731, 0.895
No. of measured, independent and observed [*I* > 2σ(*I*)] reflections	37156, 4975, 4880
*R*_int_	0.034
(sin θ/λ)_max_ (Å^−1^)	0.768

Refinement	
*R*[*F*^2^ > 2σ(*F*^2^)], *wR*(*F*^2^), *S*	0.029, 0.064, 1.11
No. of reflections	4975
No. of parameters	226
No. of restraints	2
H-atom treatment	All H-atom parameters refined
Δρ_max_, Δρ_min_ (e Å^−3^)	0.89, −0.87
Absolute structure	Flack *x* determined using 1967 quotients [(*I*^+^)−(*I*^−^)]/[(*I*^+^)+(*I*^−^)] (Parsons *et al.*, 2013 ▸)
Absolute structure parameter	0.008 (3)

Computer programs: *APEX2* and *SAINT* (Bruker, 2014 ▸), *SHELXT* (Sheldrick, 2015*a* ▸), *SHELXL2013* (Sheldrick, 2015*b* ▸).

## supplementary crystallographic information

### 2-Oxo-2H-chromen-4-yl 4-bromobenzoate Crystal data

**Table:**

C_16_H_9_BrO_4_	*D*_x_ = 1.736 Mg m^−^^3^
*M**_r_* = 345.14	Mo *K*α radiation, λ = 0.71073 Å
Orthorhombic, *P*2_1_2_1_2_1_	Cell parameters from 4940 reflections
*a* = 5.4003 (11) Å	θ = 2.3–30.6°
*b* = 6.367 (2) Å	µ = 3.13 mm^−^^1^
*c* = 38.404 (8) Å	*T* = 100 K
*V* = 1320.4 (6) Å^3^	Prism, colourless
*Z* = 4	0.27 × 0.21 × 0.13 mm
*F*(000) = 688	

### 2-Oxo-2H-chromen-4-yl 4-bromobenzoate Data collection

**Table:**

Bruker D8 Venture diffractometer	4880 reflections with *I* > 2σ(*I*)
φ and ω scans	*R*_int_ = 0.034
Absorption correction: multi-scan (SADBAS; Krause *et al.*, 2015)	θ_max_ = 33.1°, θ_min_ = 3.2°
*T*_min_ = 0.731, *T*_max_ = 0.895	*h* = −7→8
37156 measured reflections	*k* = −9→9
4975 independent reflections	*l* = −58→58

### 2-Oxo-2H-chromen-4-yl 4-bromobenzoate Refinement

**Table:**

Refinement on *F*^2^	Hydrogen site location: difference Fourier map
Least-squares matrix: full	All H-atom parameters refined
*R*[*F*^2^ > 2σ(*F*^2^)] = 0.029	*w* = 1/[σ^2^(*F*_o_^2^) + (0.0042*P*)^2^ + 1.5505*P*] where *P* = (*F*_o_^2^ + 2*F*_c_^2^)/3
*wR*(*F*^2^) = 0.064	(Δ/σ)_max_ = 0.001
*S* = 1.11	Δρ_max_ = 0.89 e Å^−^^3^
4975 reflections	Δρ_min_ = −0.87 e Å^−^^3^
226 parameters	Absolute structure: Flack *x* determined using 1967 quotients [(*I*^+^)-(*I*^-^)]/[(*I*^+^)+(*I*^-^)] (Parsons *et al.*, 2013)
2 restraints	Absolute structure parameter: 0.008 (3)

### 2-Oxo-2H-chromen-4-yl 4-bromobenzoate Special details

**Table:**

Geometry. All esds (except the esd in the dihedral angle between two l.s. planes) are estimated using the full covariance matrix. The cell esds are taken into account individually in the estimation of esds in distances, angles and torsion angles; correlations between esds in cell parameters are only used when they are defined by crystal symmetry. An approximate (isotropic) treatment of cell esds is used for estimating esds involving l.s. planes.

### 2-Oxo-2H-chromen-4-yl 4-bromobenzoate Fractional atomic coordinates and isotropic or equivalent isotropic displacement parameters (Å^2^)

**Table:**

	*x*	*y*	*z*	*U*_iso_*/*U*_eq_	
Br1	0.42360 (5)	−0.17161 (4)	0.27424 (2)	0.02298 (6)	
C5	0.3903 (4)	0.3282 (4)	0.34326 (6)	0.0189 (4)	
O2	0.2448 (3)	0.6887 (3)	0.37643 (4)	0.0196 (3)	
C2	0.0989 (5)	0.1701 (5)	0.28876 (6)	0.0209 (4)	
O3	−0.1051 (4)	1.3465 (3)	0.41125 (5)	0.0301 (4)	
C8	0.2098 (5)	0.8636 (4)	0.39677 (6)	0.0173 (4)	
O4	0.2035 (4)	1.2090 (3)	0.44169 (5)	0.0231 (4)	
O1	−0.1096 (4)	0.7132 (3)	0.34419 (6)	0.0291 (5)	
C7	0.0804 (6)	0.6245 (4)	0.35106 (6)	0.0189 (4)	
C3	0.0272 (4)	0.3517 (4)	0.30635 (6)	0.0192 (4)	
C6	0.4630 (5)	0.1452 (4)	0.32592 (6)	0.0188 (4)	
C15	0.5666 (5)	0.7219 (4)	0.43182 (6)	0.0190 (4)	
C4	0.1718 (5)	0.4307 (4)	0.33372 (6)	0.0175 (4)	
C9	0.0415 (5)	1.0175 (4)	0.39187 (7)	0.0209 (5)	
C12	0.5369 (5)	1.0775 (4)	0.47484 (7)	0.0225 (5)	
C11	0.3749 (5)	1.0522 (4)	0.44687 (6)	0.0183 (4)	
C10	0.0358 (5)	1.1996 (4)	0.41461 (7)	0.0219 (5)	
C16	0.3865 (5)	0.8749 (4)	0.42525 (6)	0.0162 (4)	
C14	0.7299 (5)	0.7462 (4)	0.45953 (7)	0.0217 (5)	
C1	0.3163 (5)	0.0707 (4)	0.29888 (6)	0.0179 (4)	
C13	0.7138 (5)	0.9227 (5)	0.48089 (7)	0.0228 (5)	
H15	0.579 (7)	0.600 (5)	0.4167 (8)	0.019 (8)*	
H9	−0.096 (8)	1.018 (6)	0.3743 (9)	0.029 (9)*	
H2	−0.004 (4)	0.106 (4)	0.2698 (6)	0.026 (9)*	
H13	0.823 (6)	0.936 (5)	0.5001 (9)	0.021 (9)*	
H3	−0.124 (6)	0.423 (5)	0.2999 (8)	0.015 (8)*	
H12	0.518 (6)	1.206 (6)	0.4896 (9)	0.027 (9)*	
H6	0.597 (7)	0.074 (5)	0.3328 (8)	0.016 (8)*	
H14	0.851 (7)	0.640 (6)	0.4629 (9)	0.026 (9)*	
H5	0.492 (7)	0.386 (6)	0.3636 (10)	0.037 (11)*	

### 2-Oxo-2H-chromen-4-yl 4-bromobenzoate Atomic displacement parameters (Å^2^)

**Table:**

	*U*^11^	*U*^22^	*U*^33^	*U*^12^	*U*^13^	*U*^23^
Br1	0.02724 (11)	0.02177 (10)	0.01994 (10)	0.00231 (11)	−0.00180 (10)	−0.00603 (9)
C5	0.0192 (10)	0.0202 (10)	0.0172 (9)	−0.0005 (10)	−0.0020 (8)	−0.0020 (9)
O2	0.0223 (8)	0.0163 (8)	0.0201 (8)	0.0048 (7)	−0.0044 (6)	−0.0035 (7)
C2	0.0224 (11)	0.0223 (10)	0.0181 (9)	0.0002 (12)	−0.0044 (8)	0.0000 (9)
O3	0.0362 (11)	0.0234 (9)	0.0308 (9)	0.0166 (10)	0.0030 (8)	−0.0011 (8)
C8	0.0202 (10)	0.0147 (10)	0.0169 (9)	0.0023 (8)	0.0016 (8)	0.0000 (7)
O4	0.0279 (9)	0.0174 (9)	0.0238 (8)	0.0066 (7)	0.0018 (7)	−0.0035 (7)
O1	0.0261 (10)	0.0266 (10)	0.0345 (10)	0.0087 (8)	−0.0092 (8)	−0.0063 (8)
C7	0.0213 (10)	0.0174 (10)	0.0182 (9)	0.0006 (9)	−0.0019 (9)	0.0013 (7)
C3	0.0190 (10)	0.0192 (11)	0.0194 (9)	0.0003 (9)	−0.0020 (8)	0.0010 (8)
C6	0.0185 (11)	0.0193 (11)	0.0185 (9)	0.0014 (9)	−0.0014 (8)	−0.0014 (8)
C15	0.0213 (10)	0.0177 (10)	0.0180 (9)	0.0037 (9)	−0.0007 (9)	−0.0007 (7)
C4	0.0199 (10)	0.0177 (10)	0.0148 (9)	−0.0009 (9)	−0.0008 (8)	−0.0002 (8)
C9	0.0231 (12)	0.0187 (10)	0.0210 (10)	0.0073 (9)	0.0002 (9)	0.0000 (8)
C12	0.0266 (14)	0.0215 (11)	0.0195 (10)	−0.0011 (10)	0.0015 (9)	−0.0045 (9)
C11	0.0207 (12)	0.0162 (10)	0.0181 (10)	0.0020 (8)	0.0019 (8)	−0.0005 (8)
C10	0.0248 (12)	0.0177 (11)	0.0231 (10)	0.0057 (9)	0.0043 (9)	0.0003 (8)
C16	0.0190 (11)	0.0144 (9)	0.0150 (9)	0.0017 (8)	0.0021 (8)	−0.0002 (7)
C14	0.0228 (11)	0.0225 (11)	0.0199 (11)	0.0037 (10)	−0.0014 (9)	0.0002 (9)
C1	0.0216 (11)	0.0178 (10)	0.0141 (9)	−0.0010 (9)	0.0029 (8)	−0.0009 (8)
C13	0.0242 (12)	0.0247 (12)	0.0195 (11)	−0.0020 (10)	−0.0020 (9)	−0.0015 (9)

### 2-Oxo-2H-chromen-4-yl 4-bromobenzoate Geometric parameters (Å, º)

**Table:**

Br1—C1	1.900 (3)	C3—C4	1.403 (3)
C5—C4	1.397 (4)	C3—H3	0.97 (3)
C5—C6	1.398 (4)	C6—C1	1.389 (3)
C5—H5	1.02 (4)	C6—H6	0.89 (4)
O2—C8	1.373 (3)	C15—C14	1.391 (4)
O2—C7	1.380 (3)	C15—C16	1.400 (3)
C2—C1	1.389 (4)	C15—H15	0.97 (3)
C2—C3	1.394 (4)	C9—C10	1.452 (4)
C2—H2	1.002 (12)	C9—H9	1.00 (4)
O3—C10	1.213 (3)	C12—C13	1.392 (4)
C8—C9	1.350 (3)	C12—C11	1.394 (4)
C8—C16	1.453 (3)	C12—H12	1.00 (4)
O4—C11	1.376 (3)	C11—C16	1.403 (3)
O4—C10	1.380 (3)	C14—C13	1.394 (4)
O1—C7	1.200 (3)	C14—H14	0.95 (4)
C7—C4	1.487 (3)	C13—H13	0.95 (3)
			
C4—C5—C6	120.1 (2)	C3—C4—C7	116.6 (2)
C4—C5—H5	119 (2)	C8—C9—C10	120.7 (2)
C6—C5—H5	121 (2)	C8—C9—H9	127 (2)
C8—O2—C7	123.6 (2)	C10—C9—H9	113 (2)
C1—C2—C3	118.5 (2)	C13—C12—C11	118.5 (2)
C1—C2—H2	119.2 (9)	C13—C12—H12	124 (2)
C3—C2—H2	122.3 (9)	C11—C12—H12	118 (2)
C9—C8—O2	127.0 (2)	O4—C11—C12	116.7 (2)
C9—C8—C16	120.7 (2)	O4—C11—C16	121.9 (2)
O2—C8—C16	112.2 (2)	C12—C11—C16	121.4 (2)
C11—O4—C10	121.3 (2)	O3—C10—O4	117.3 (2)
O1—C7—O2	124.5 (2)	O3—C10—C9	124.4 (3)
O1—C7—C4	125.2 (2)	O4—C10—C9	118.3 (2)
O2—C7—C4	110.4 (2)	C15—C16—C11	119.0 (2)
C2—C3—C4	120.4 (2)	C15—C16—C8	123.9 (2)
C2—C3—H3	120.1 (19)	C11—C16—C8	117.2 (2)
C4—C3—H3	119.5 (19)	C15—C14—C13	120.0 (3)
C1—C6—C5	118.7 (2)	C15—C14—H14	118 (2)
C1—C6—H6	120 (2)	C13—C14—H14	122 (2)
C5—C6—H6	121 (2)	C2—C1—C6	122.4 (2)
C14—C15—C16	120.1 (2)	C2—C1—Br1	119.22 (19)
C14—C15—H15	120 (2)	C6—C1—Br1	118.39 (19)
C16—C15—H15	120 (2)	C12—C13—C14	121.0 (3)
C5—C4—C3	119.9 (2)	C12—C13—H13	120 (2)
C5—C4—C7	123.4 (2)	C14—C13—H13	119 (2)
			
C7—O2—C8—C9	−12.3 (4)	C11—O4—C10—C9	1.0 (4)
C7—O2—C8—C16	169.9 (2)	C8—C9—C10—O3	177.9 (3)
C8—O2—C7—O1	−0.3 (4)	C8—C9—C10—O4	−1.3 (4)
C8—O2—C7—C4	179.9 (2)	C14—C15—C16—C11	0.3 (4)
C1—C2—C3—C4	−0.3 (4)	C14—C15—C16—C8	179.5 (2)
C4—C5—C6—C1	1.1 (4)	O4—C11—C16—C15	179.5 (2)
C6—C5—C4—C3	−1.0 (4)	C12—C11—C16—C15	−0.7 (4)
C6—C5—C4—C7	179.4 (2)	O4—C11—C16—C8	0.3 (3)
C2—C3—C4—C5	0.6 (4)	C12—C11—C16—C8	−179.9 (2)
C2—C3—C4—C7	−179.8 (2)	C9—C8—C16—C15	−179.7 (2)
O1—C7—C4—C5	−177.8 (3)	O2—C8—C16—C15	−1.8 (3)
O2—C7—C4—C5	2.0 (3)	C9—C8—C16—C11	−0.6 (4)
O1—C7—C4—C3	2.5 (4)	O2—C8—C16—C11	177.3 (2)
O2—C7—C4—C3	−177.7 (2)	C16—C15—C14—C13	0.2 (4)
O2—C8—C9—C10	−176.5 (2)	C3—C2—C1—C6	0.4 (4)
C16—C8—C9—C10	1.0 (4)	C3—C2—C1—Br1	−177.87 (19)
C10—O4—C11—C12	179.6 (2)	C5—C6—C1—C2	−0.8 (4)
C10—O4—C11—C16	−0.6 (4)	C5—C6—C1—Br1	177.48 (19)
C13—C12—C11—O4	−179.7 (2)	C11—C12—C13—C14	0.1 (4)
C13—C12—C11—C16	0.5 (4)	C15—C14—C13—C12	−0.5 (4)
C11—O4—C10—O3	−178.2 (2)		

### 2-Oxo-2H-chromen-4-yl 4-bromobenzoate Hydrogen-bond geometry (Å, º)

**Table:**

*D*—H···*A*	*D*—H	H···*A*	*D*···*A*	*D*—H···*A*
C9—H9···O1	1.00 (4)	2.26 (4)	2.788 (3)	111 (3)
C15—H15···O3^i^	0.97 (3)	2.36 (4)	3.079 (3)	131 (3)

Symmetry code: (i) *x*+1, *y*−1, *z*.
